# The General Anesthetic Isoflurane Bilaterally Modulates Neuronal Excitability

**DOI:** 10.1016/j.isci.2019.100760

**Published:** 2019-12-10

**Authors:** Mengchan Ou, Wenling Zhao, Jin Liu, Peng Liang, Han Huang, Hai Yu, Tao Zhu, Cheng Zhou

**Affiliations:** 1 Laboratory of Anesthesia & Critical Care Medicine, Translational Neuroscience Center, West China Hospital of Sichuan University, Chengdu 610041, Sichuan, P.R. China; 2 Department of Anesthesiology, West China Hospital of Sichuan University, Chengdu 610041, Sichuan, P.R. China; 3 Department of Anesthesiology, West China Second Hospital of Sichuan University, Chengdu 610041, Sichuan, P.R. China

**Keywords:** Anesthesiology, Neuroscience

## Abstract

Volatile anesthetics induce hyperactivity during induction while producing anesthesia at higher concentrations. They also bidirectionally modulate many neuronal functions. However, the neuronal mechanism is unclear. The effects of isoflurane on sodium channel currents were analyzed in acute mouse brain slices, including sodium leak (NALCN) currents and voltage-gated sodium channels (Na_v_) currents. Isoflurane at sub-anesthetic concentrations increased the spontaneous firing rate of CA3 pyramidal neurons, whereas anesthetic concentrations of isoflurane decreased the firing rate. Isoflurane at sub-anesthetic concentrations enhanced NALCN conductance but minimally inhibited Na_v_ currents. Isoflurane at anesthetic concentrations depressed Na_v_ currents and action potential amplitudes. Isoflurane at sub-anesthetic concentrations depolarized resting membrane potential (RMP) of neurons, whereas hyperpolarized the RMP at anesthetic concentrations. Isoflurane at low concentrations induced hyperactivity *in vivo*, which was diminished in NALCN knockdown mice. In conclusion, enhancement of NALCN by isoflurane contributes to its bidirectional modulation of neuronal excitability and the hyperactivity during induction.

## Highlights

• Volatile anesthetic isoflurane exerts bidirectional modulation of neuronal excitability
• Isoflurane enhances NALCN conductance at sub-anesthetic concentration
• NALCN knockdown diminishes behavioral hyperactivity during isoflurane induction

## Graphical Abstract

**Figure:**
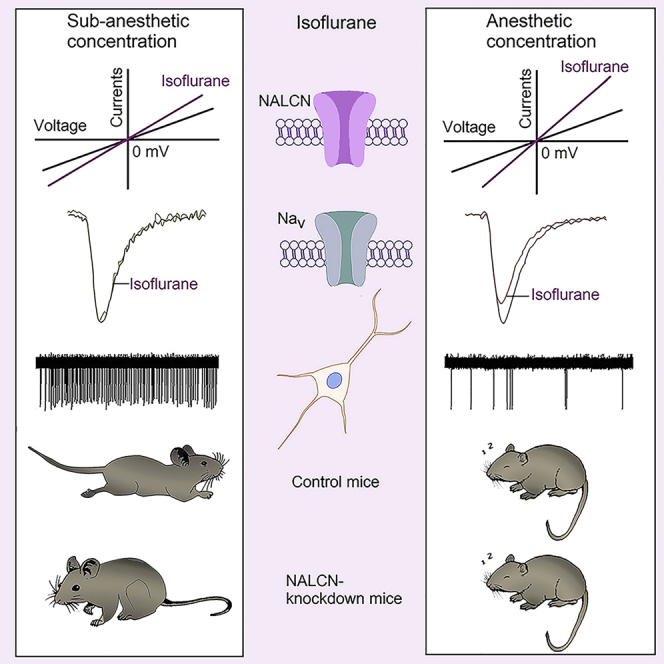

## Introduction

General anesthetics disrupt the balance of inhibitory and excitatory neurotransmission to produce widespread depression in the central nervous system (Becker et al., 2012, Clark and Rosner, 1973, Leung et al., 2014, Palanca et al., 2017, Son, 2010). Volatile anesthetics are widely used general anesthetics, which exhibit more complex mechanisms than intravenous general anesthetics, as they interact with multiple molecular targets (Franks, 2008, Hemmings et al., 2005).

Volatile anesthetics (e.g. isoflurane and sevoflurane) can induce hyperactivity during the mask induction and recovery stages of general anesthesia (Costi et al., 2014, Liang et al., 2017) but result in unconsciousness and immobility at higher concentrations (Sonner et al., 2003). It has been well documented that general anesthetics produce more than a simple depression in the mammalian central nervous system and can even produce excitatory activities (MacIver and Roth, 1987, MacIver and Roth, 1988). Isoflurane increased hippocampal CA1 neuronal excitability at sub-anesthetic concentration and produced postsynaptic depression of dentate neurons at anesthetic concentrations (MacIver and Roth, 1988). Specific volatile anesthetics such as enflurane can even induce seizures (Sleigh et al., 2009, Voss et al., 2008). These outcomes indicate that volatile anesthetics may bidirectionally modulate neuronal excitability. Diverse neuronal functions, including synaptic transmission and plasticity, have been found to be bidirectionally modulated by volatile anesthetics (MacIver, 2014, Ogawa et al., 2011, Otsubo et al., 2008, Xiao et al., 2016). However, it is unclear how volatile anesthetics exert bidirectional modulation of neuronal excitability. Understanding the molecular targets is important for attenuating the unwanted hyperactivities induced by volatile anesthetics during induction of general anesthesia. Stabilized maintenance and recovery can improve quality of general anesthesia and safety of patients throughout.

Sodium channels are important for determining the neuronal excitability and action potential (AP) discharge in the CNS (Eijkelkamp et al., 2012, Wang et al., 2017). Volatile anesthetics at clinically relevant concentrations inhibit voltage-gated sodium channel (Na_v_) currents in transfected cells (Herold et al., 2009, Yamada-Hanff and Bean, 2015, Zhou et al., 2019), nerve terminals (Ouyang et al., 2003, Yamada-Hanff and Bean, 2015), dorsal root ganglia (DRG) (Scholz et al., 1998, Zhou et al., 2011), and hippocampal neurons (Zhao et al., 2019). Isoflurane reduces AP amplitude and frequency (Wu et al., 2004, Zhao et al., 2019), which may also be mediated by inhibition of sodium channels.

The voltage-independent sodium leak channel (NALCN) is widely expressed, producing a small background leak Na^+^ current at the resting membrane potential and regulating neuronal excitability (Cochet-Bissuel et al., 2014, Ford et al., 2018, Lu et al., 2010). NACLN contributes to many physiological processes, including respiratory rhythms (Lu et al., 2007, Shi et al., 2016). Isoflurane enhances the excitability of retrotrapezoid nucleus (RTN) neurons, which may be partly contributed by background sodium conductance (Lazarenko et al., 2010). However, it is unclear whether volatile anesthetics can modulate NALCN.

The hippocampus is critical for many brain functions, which are sensitive to the actions of general anesthetics (Lee et al., 2014a, Lee et al., 2014b, Zhong et al., 2014), including learning and memory (Moser and Moser, 1998). Pyramidal neurons are the principal excitatory neurons in the hippocampus and express multiple types of sodium channels (Hotson et al., 1979). We therefore designed this study to determine whether the volatile anesthetic isoflurane can bidirectionally modulate hippocampal pyramidal neurons by targeting NALCN and Na_v_and whether silencing NALCN can diminish isoflurane-induced hyperactivity during anesthesia induction *in vivo*.

## Results

### NALCN Is Widely Expressed in the Cortical and Hippocampal Neurons

**Figure 1:**
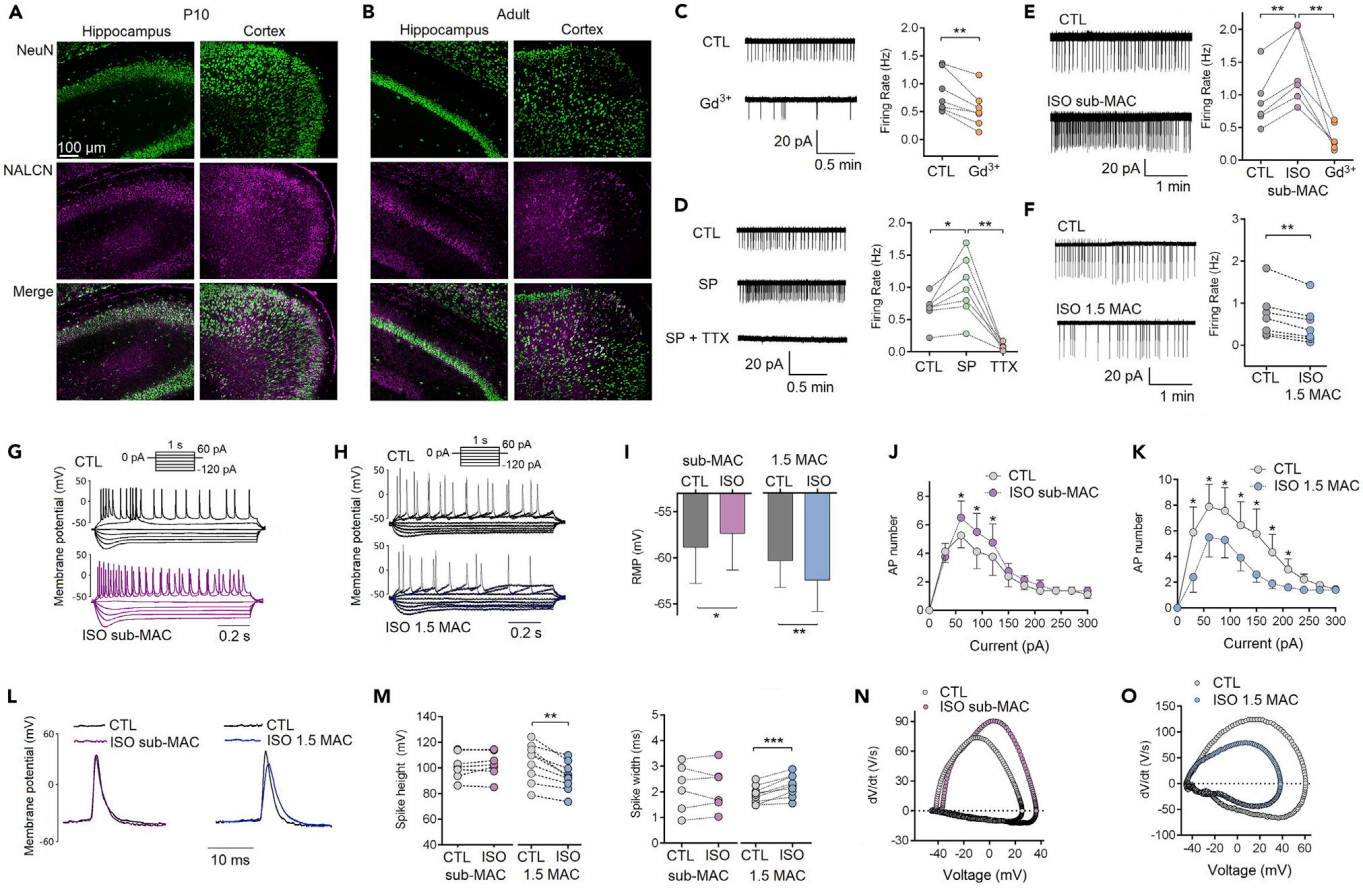
Isoflurane Bidirectionally Modulates the Excitability of Hippocampal CA3 Pyramidal Neurons (A and B) The expressional profiles of NALCN channel in hippocampus and cortex of mice were detected by immunofluorescence staining at age of postnatal day 10 (P10) (A) and adult age (B). NALCN channels are widely expressed in neurons throughout these brain regions at both ages. (C) Cell-attached mode was used to record firing rate under control (CTL) or Gd^3+^ (50 μM) conditions. (D) Firing rate of hippocampal CA3 pyramidal neuron was increased by substance P (SP, 10 μM) and diminished by tetrodotoxin (TTX, 500 nM). (E) Representative traces (left) and quantification (right) for firing rate under control or sub-MAC (0.4–0.5 MAC, 0.12–0.15 mM) isoflurane (ISO) conditions. (F) Representative traces (left) and quantification (right) for firing rate under control or ∼1.5 MAC (0.4–0.5 mM) isoflurane conditions. (G and H) Representative traces of evoked action potentials (APs). (I) Quantification for resting membrane potential (RMP) by sub-anesthetic isoflurane (left) and anesthetic concentration of isoflurane (right). (J and K) Numbers of APs were increased by sub-MAC isoflurane (J) and decreased by 1.5 MAC isoflurane (K), respectively. (L) Representative traces of the effects of isoflurane at two concentrations (sub-MAC, ∼1.5 MAC) on single APs. (M) AP height (left) was reduced and AP width (right) increased by 1.5 MAC isoflurane but not by sub-MAC isoflurane. (N and O) The effects of isoflurane on AP dynamics were illustrated by *dV/dt* analysis. Data are mean ± SEM. *p < 0.05, **p < 0.01, ***p < 0.001. Results were compared by paired t test.

NALCN was widely expressed throughout cortex and hippocampus (Figures 1A and 1B). NALCN contributes to the firing rate of hippocampal CA3 pyramidal neurons. Exposure to Gd^3+^ (50 μM) decreased the firing rate from 0.85 ± 0.14 to 0.54 ± 0.12 Hz (p = 0.003, n = 7; Figure 1C), whereas substance P (SP, 10 μM) caused an increase in firing rate from 0.67 ± 0.09 to 1.00 ± 0.18 Hz (p = 0.032, n = 7; Figure 1D). Notably, tetrodotoxin (TTX, 500 nM) diminished the firing rate even when neurons were exposed to SP (p = 0.001, n = 7; Figure 1D), indicating that inhibition of TTX-S Na_v_ depresses neuronal excitability, even while NALCN channels are activated.

### Isoflurane Produces Bidirectional Actions on Neuronal Excitability

Isoflurane at sub-anesthetic concentrations (0.12–0.15 mM, ∼0.4–0.5 MAC) increased the spontaneous firing rate from 0.90 ± 0.17 to 1.38 ± 0.22 Hz (p = 0.008, n = 6; Figure 1E), whereas ∼1.5 MAC (0.4–0.5 mM) decreased the firing rate from 0.72 ± 0.21 to 0.50 ± 0.18 Hz (p = 0.001, n = 7; Figure 1F). Gd^3+^ (50 μM) abolished the increased firing rate induced by isoflurane (Figure 1E). Sub-anesthetic concentration of isoflurane depolarized the resting membrane potential (RMP) of neurons from −58.84 ± 1.40 to −57.34 ± 1.41 mV (p = 0.006, n = 8; Figure 1I), whereas ∼1.5 MAC isoflurane hyperpolarized the RMP from −60.35 ± 2.91 to −62.47 ± 3.40 mV (p = 0.015, n = 6; Figure 1I).

### Isoflurane Depresses Action Potentials at Anesthetic Concentrations

Discharge of APs was activated by 1s series current injection from −120 to 60 pA (Figures 1G and 1H). Sub-anesthetic isoflurane increased the numbers of APs (Figure 1J). Isoflurane at ∼1.5 MAC reduced APs frequency (Figure 1K). Single AP was evoked by injection of a 60-pA current for 100 ms (Figure 1L). Isoflurane at 1.5 MAC reduced AP height from 104.44 ± 4.89 to 93.24 ± 3.69 mV (p < 0.001, n = 9; Figure 1M) and increased AP width from 1.89 ± 0.11 to 2.24 ± 0.14 ms (p < 0.001, n = 9). Isoflurane at sub-anesthetic concentrations did not depress single AP. By phase-plane plot *dV*/*dt* analysis, isoflurane at sub-anesthetic and 1.5 MAC showed the opposite effects on the whole-time course of spikes (Figures 1N and 1O).

### Isoflurane Enhances NALCN Channel Currents in Acute Brain Slices

**Figure 2:**
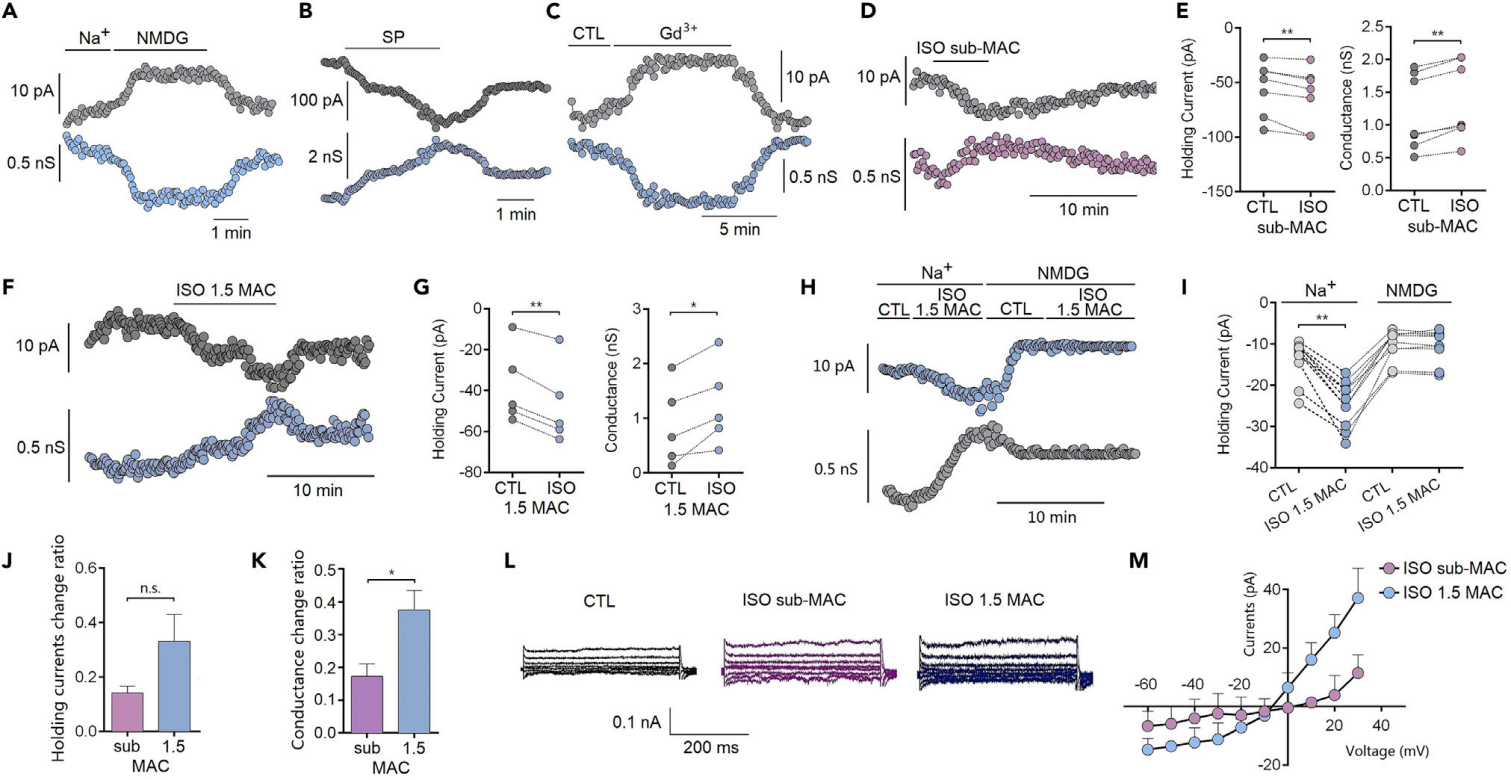
Isoflurane Enhances NALCN in Hippocampal CA3 Pyramidal Neuron at Sub-anesthetic Concentrations (A–C) Whole-cell voltage-clamp recording (V_holding_ = −60 mV) of holding current (top) and conductance (bottom) from hippocampal CA3 pyramidal neurons in the conditions that replacing extracellular Na^+^ with NMDG (A), perfusion with substance P (SP, 10 μM) (B), or Gd^3+^ (50 μM) (C), respectively. (D and E) Representative traces (D) and statistical analysis (E) of holding currents and conductance of CA3 pyramidal neurons’ exposure to sub-anesthetic isoflurane (0.12–0.15 mM). (F and G) Representative traces (F) and statistical analysis (G) of holding currents and conductance of CA3 pyramidal neurons’ exposure to 1.5 MAC (0.4–0.5 mM) isoflurane. (H and I) Isoflurane at 1.5 MAC (0.4–0.5 mM) did not increase the holding currents after replacing extracellular Na^+^ with NMDG. (J and K) The percentage change of holding current (J) and conductance (K) between sub-MAC (0.12–0.15 mM, 0.4–0.5 MAC) and ∼1.5-MAC (0.4–0.5 mM) isoflurane. (L) Representative traces of currents were evoked by step voltage pulses (−60 to +30 mV). (M) Current-voltage relationship of isoflurane-sensitive currents (n = 5–6). Data are mean ± SEM. *p < 0.05, **p < 0.01. Results were compared by paired t test (E, G, and I) or unpaired t test (J and K).

To record NALCN channel currents, 25 mM TEA-Cl, 500 nM TTX, and 10 μM CNQX were added to the bath solution. With the holding potential at −60 mV and extracellular Na^+^ replaced with NMDG, holding currents were significantly decreased by 53.72% ± 12.56% (Figure 2A top), indicating that Na^+^ contributes to these leak currents (Figure 2A bottom). These holding currents were significantly activated by SP (10 μM) and inhibited by Gd^3+^ (50 μM) (Figures 2B and 2C). Isoflurane at both sub-anesthetic and ∼1.5 MAC increased the holding currents from −55.40 ± 9.20 to −63.01 ± 10.15 pA (p = 0.006, n = 7; Figure 2E left) and from −37.98 ± 8.35 to −47.27 ± 8.79 pA (p < 0.001, n = 5; Figure 2G left), respectively. The channel conductance correspondingly increased from 1.18 ± 0.22 to 1.35 ± 0.23 nS (p < 0.001, n = 7; Figure 2E right) and 0.86 ± 0.33 to 1.24 ± 0.34 nS (p = 0.016, n = 5; Figure 2G right), respectively. The enhancement of holding currents by isoflurane was reduced when extracellular Na^+^ replaced with NMDG (Figure 2H). Isoflurane at anesthetic concentrations showed greater effects compared with sub-anesthetic concentrations (Figures 2J and 2K). The I-V curves indicated a voltage-independent current with a reversal potential near 0 mV (Figures 2L and 2M).

### Isoflurane Does Not Enhance NALCN Channel Conductance *per se* in Transfected HEK Cells

**Figure 3:**
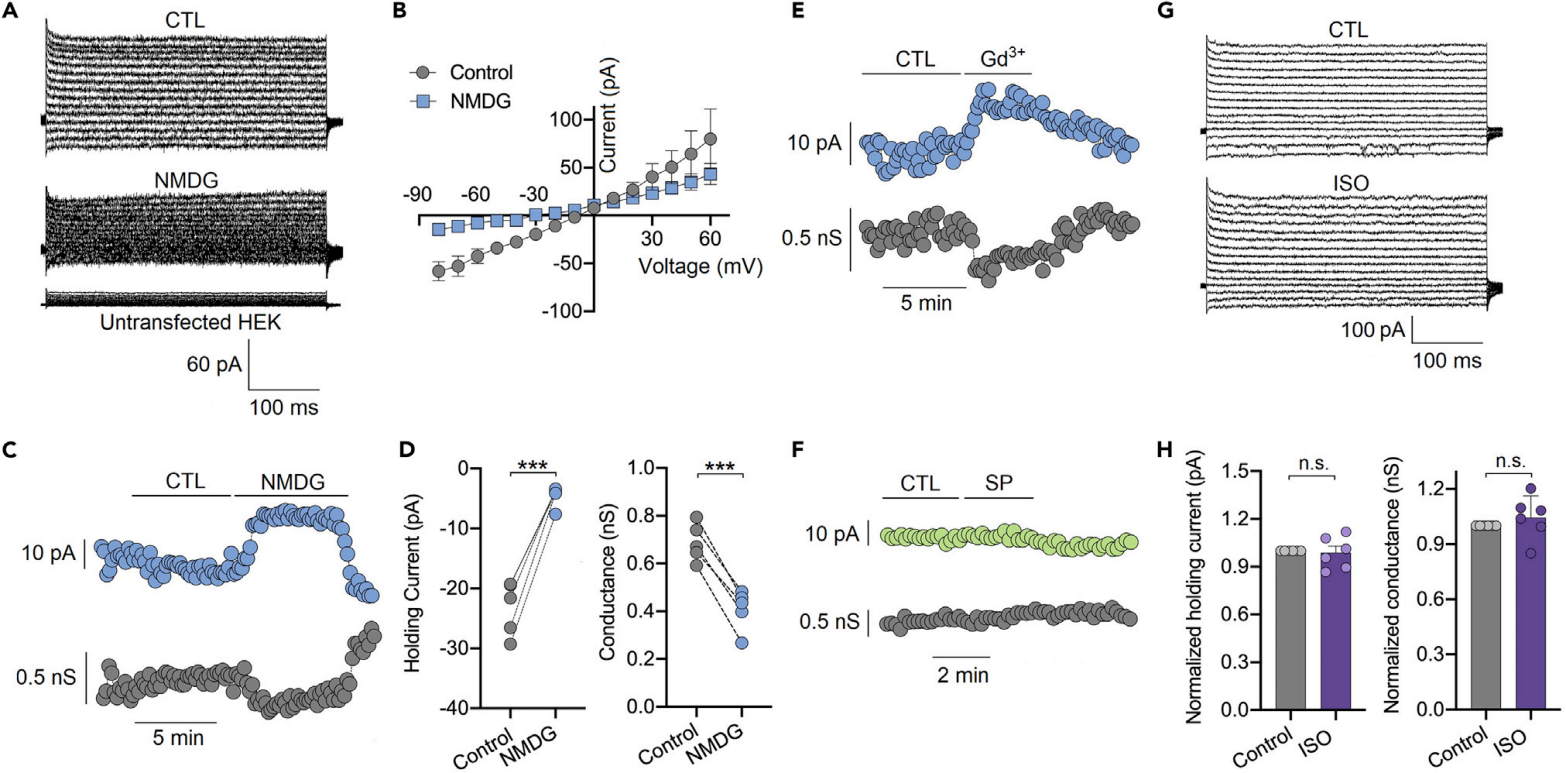
Isoflurane Does Not Enhance NALCN Channel Conductance *per se* in Transfected HEK Cells The effects of isoflurane on NALCN channel currents were recorded in NALCN channel transfected HEK cells. (A) The representative traces of NALCN channel currents recorded in transfected HEK cells. The currents were diminished after replacing extracellular Na^+^ with NMDG. (B) Current-voltage relationship of NALCN-mediated currents (n = 4). (C) Whole-cell voltage-clamp recording (V_holding_ = −60 mV) of holding current (top) and conductance (bottom) in the conditions that replacing extracellular Na^+^ with NMDG. (D) Statistical analysis of the holding current (left) and conductance (right) in the conditions that replacing extracellular Na^+^ with NMDG (n = 5). (E and F) Whole-cell voltage-clamp recording (V_holding_ = −60 mV) of holding current (top) and conductance (bottom) in the conditions that perfusion with Gd^3+^ (50 μM) (E) and/or substance P (SP, 10 μM) (F), respectively. SP did not enhance NALCN channel *per se* (F). (G) The representative traces of NALCN channel currents recorded in transfected HEK cells before and after perfusion of ∼1.5 MAC (0.4–0.5 mM) isoflurane. (H) Isoflurane at ∼1.5 MAC did not enhance NALCN-mediated leak currents (left) and channel conductance (right) (n = 6). Data are mean ± SEM. ***p < 0.001. Results were compared by paired t test.

To determine whether isoflurane directly enhance NALCN channel *per se* or through NALCN complex or other mechanisms, the effects of isoflurane on NALCN channel currents were recorded in NALCN channel transfected HEK cells (Figure 3A). The transfected channel currents were characterized by permission of Na^+^ and voltage independent (n = 4, Figure 3B). With the holding potential at −60 mV and extracellular Na^+^ replaced with NMDG, holding currents were almost vanished (n = 5, p < 0.001, Figures 3C and 3D). These holding currents were significantly inhibited by Gd^3+^ (50 μM) (Figure 3E) but not activated by SP (10 μM) (Figure 3F). Isoflurane at ∼1.5 MAC (0.4–0.5 mM) did not enhance NALCN-mediated leak currents (Figure 3G) and produced no effect on the channel conductance (n = 6, p = 0.40, Figure 3H). These results indicate that the enhancement of isoflurane on NALCN channel currents in acute brain slices may be involved in NALCN complex (e.g. UNC79 or UNC80) or other mechanisms.

### Isoflurane Inhibits Voltage-Gated Sodium Channel Currents in Acute Brain Slices

**Figure 4:**
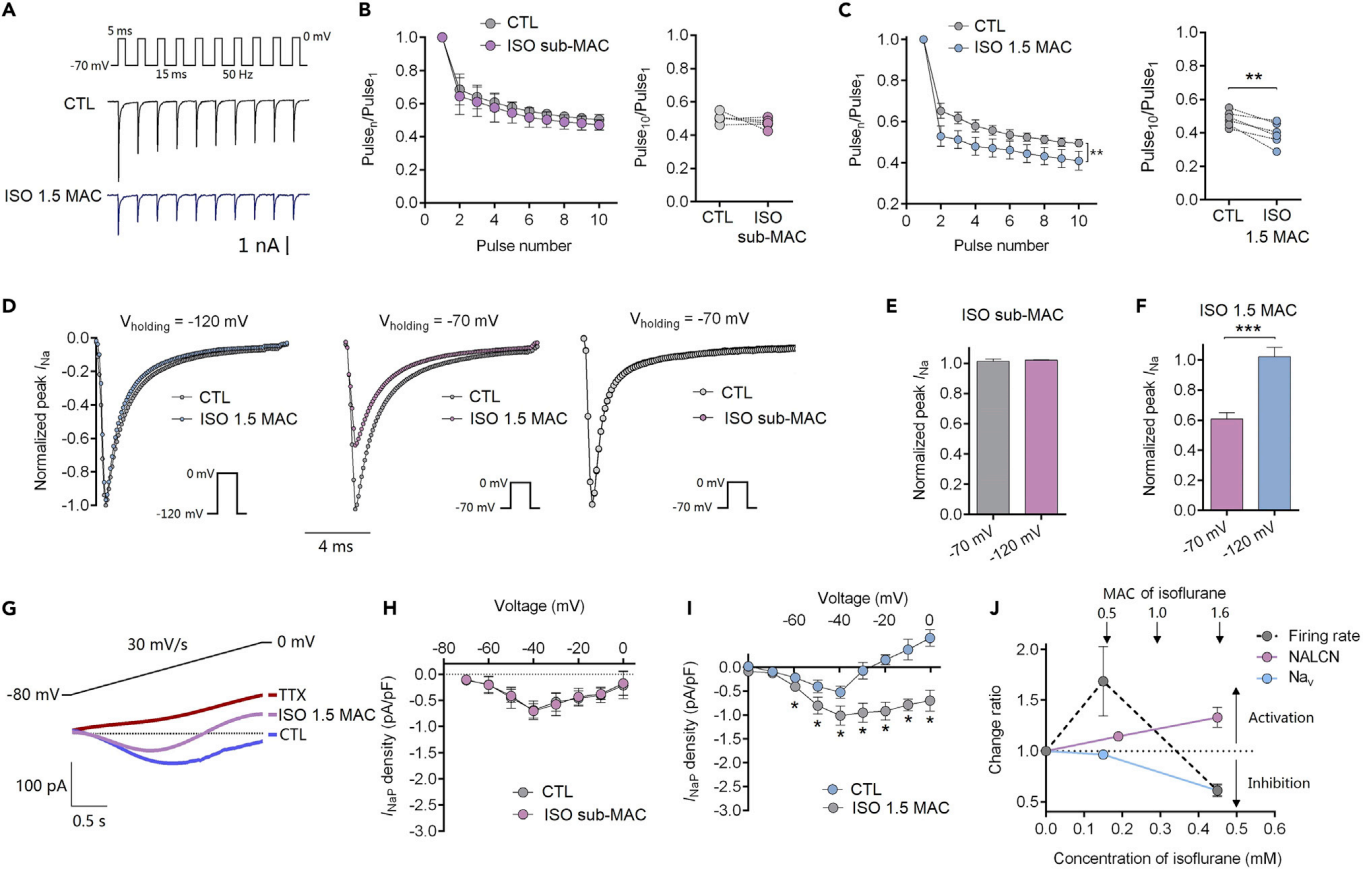
Isoflurane Activity- and Voltage-Dependently Inhibits Na_v_ Currents (A) Representative currents from the same neuron before (top) and after (bottom) isoflurane. (B and C) Isoflurane activity-dependently inhibited sodium currents at a clinically relevant concentration (0.4–0.5 mM, ∼1.5 MAC) (C), while sub-anesthetic concentration (0.12–0.15 mM, 0.4–0.5 MAC) did not (B). (D–F) The normalized sodium currents traces of pyramidal neuron before and after isoflurane application at the holding potentials of −120 mV and −70 mV (D), and the inhibition ratios showing voltage-dependent inhibition of Na_v_ currents by isoflurane (E and F). (G) Mean persistent sodium currents (*I*_NaP_) curves obtained from seven neurons, evoked with a ramp protocol from −80 mV to 0 mV (30 mV/s). (H and I) Isoflurane at 1.5 MAC (0.4–0.5 mM) significantly inhibited the density of *I*_NaP_ (I), while sub-MAC concentration did not (H). (J) Schematic of isoflurane dose-effect on firing rate, NALCN and Na_v_. Data are mean ± SEM. *p < 0.05, **p < 0.01, ***p < 0.001. Results were compared by paired t test (B, C, H, and I) or unpaired t test (E and F).

Repeated depolarizations at 50 Hz in 5-ms pulses (Figure 4A), with the *I*_Na_ of each pulse normalized to that of the first pulse (Pulse_n_/Pulse_1_), removed the effect of resting block by isoflurane. Thus, the reduced *I*_Na_ at the 10^th^ pulse reflected activity-dependent inhibition as a result of repeated membrane depolarizations. From a holding potential of −70 mV, isoflurane at 1.5 MAC reduced Pulse_10_/Pulse_1_ from 0.48 ± 0.02 to 0.40 ± 0.03 (p = 0.003, n = 6; Figure 4C), and no effect was found for sub-anesthetic isoflurane (p = 0.27, n = 5; Figure 4B). For the transient component of Na_v_ current (*I*_NaT_), which was activated at a membrane potential of 0 mV, sub-anesthetic isoflurane produced no effect (p = 0.49, n = 3; Figure 4E). At the physiologic holding potential (−70 mV), isoflurane at anesthetic concentration (∼1.5 MAC) inhibited *I*_NaT_ (60.67% ± 4.26% inhibition; n = 9; Figure 4F). However, at the holding potential of −120 mV, isoflurane exhibited little effect (Figure 4F). When persistent component of Na_v_ current (*I*_NaP_) was evoked by a ramp depolarization stimulus from −80 to 0 mV at 30 mV/s (Lunko et al., 2014), isoflurane (∼1.5 MAC) significantly decreased *I*_NaP_ densities (n = 6, Figure 4I). Isoflurane at sub-anesthetic produced little inhibition (Figure 4H). *I*_NaP_ was completely inhibited by 500 nM TTX (Figure 4G). Schematic of NALCN-Na_v_ on firing rate showed that isoflurane increased firing rate by activating NALCN and preserving Na_v_ function at sub-anesthetic concentrations, while decreased firing rates at anesthetic concentrations by inhibition of Na_v_ (Figure 4J).

### Isoflurane-induced Hyperactivity during Anesthesia Induction Is Diminished by NALCN Knockdown

**Figure 5:**
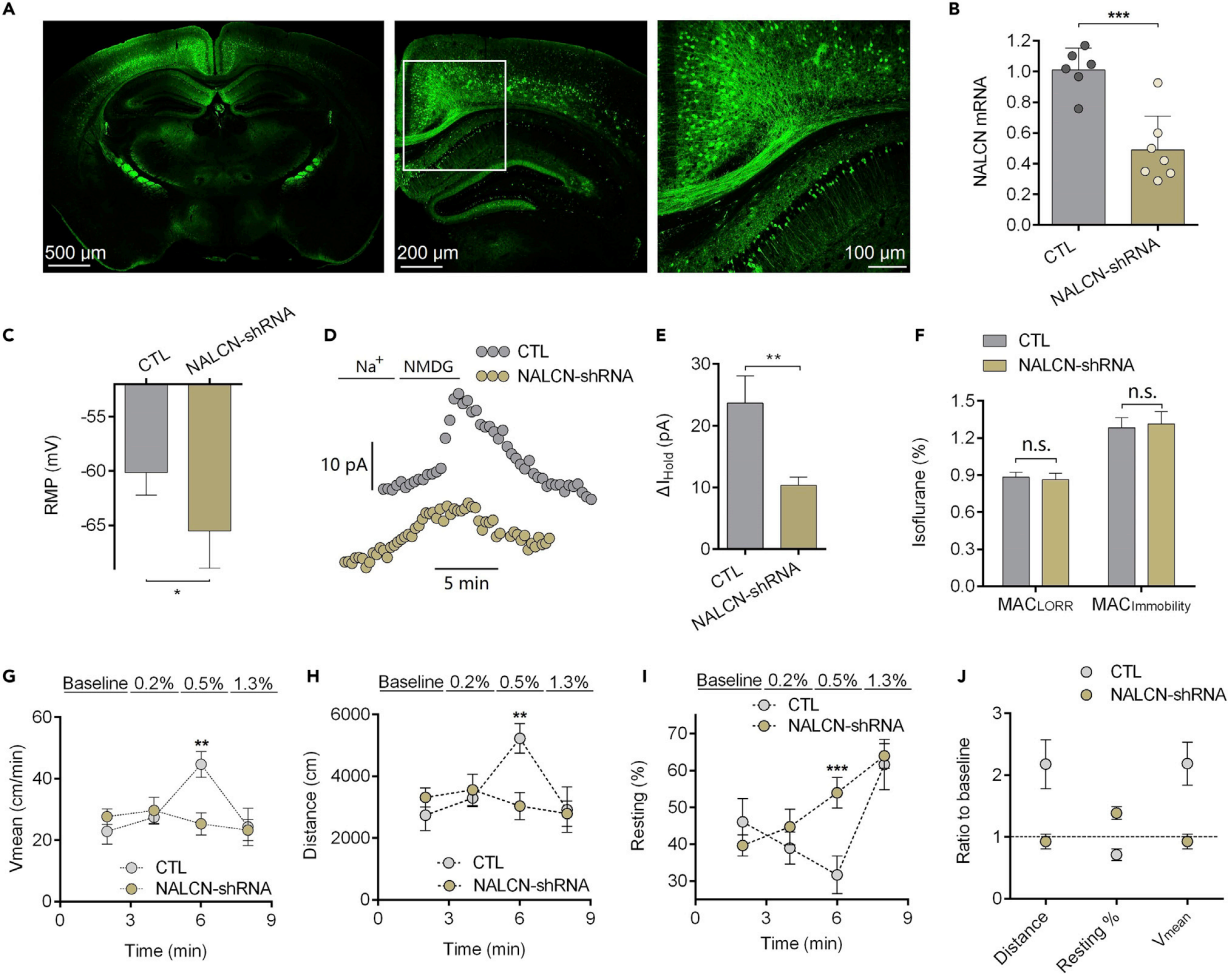
The Hyperactivity Induced by Isoflurane during Anesthesia Induction is Diminished by NALCN Knockdown (A) The representative images of AAV2-infected (GFP^+^) neurons in fixed brain slice. The right one is the enlargement of the part enclosed by a white frame in the middle image. (B) qRT-PCR analysis of NALCN mRNA in mice brain between control group and NALCN knockdown group. (C–E) Rest membrane potential (RMP) (C) and the changes of holding currents (D and E) after NALCN knockdown. (F) Minimum alveolar concentration (MAC) for loss of righting reflex (LORR) and immobility did not change between control and NALCN-shRNA-infected mice. (G–I) Measurements of mice mean speed (V_mean_) (G), total distance (Distance) (H), and resting time (Resting%) (I). (J) Ratio to baseline values of V_mean_, Distance and Resting% between control and NALCN-shRNA-infected mice under 0.5% isoflurane. Data are mean ± SEM. *p < 0.05, **p < 0.01, ***p < 0.001. Results were compared by unpaired t test.

Green fluorescent protein (GFP) was detected throughout cortical and hippocampal neurons 3–4 weeks after injection of virus (Figure 5A). NALCN mRNA level in cortex and hippocampus decreased to 48% ± 8% (p < 0.001, n = 6–7; Figure 5B) in the mice from NALCN knockdown group. Compared with control (scrambled-shRNA) mice, RMP of pyramidal neurons was hyperpolarized from −60.15 ± 2.09 to −65.87 ± 1.35 mV in NALCN knockdown mice (p = 0.0489, n = 6–7; Figure 5C). The Na^+^-mediated holding currents in the neurons from NALCN knockdown mice were smaller than that of control mice (10.33 ± 1.34 vs. 23.64 ± 4.41 pA, p = 0.006, n = 7–9; Figure 5E). Knockdown of NALCN did not change MAC of isoflurane for LORR and immobility *in vivo* (Figure 5F). Isoflurane-induced hyperactivity during induction was diminished by NALCN knockdown *in vivo*. By the behavioral test of isoflurane induction, mean speed (p = 0.002), and total distance (p = 0.005) of the mice was lower in NALCN-shRNA group than that of mice in control group at concentration of 0.5% (n = 6–8; Figures 5G, 5H, and 5J). The percentage of resting time was also higher in the mice of NALCN-shRNA group (p < 0.001, n = 6–8; Figures 5I and 5J).

## Discussion

The exact mechanisms underlying actions of general anesthetics are still unclear. General anesthetics produce widespread depression in the CNS mainly by enhancing inhibitory neurotransmission and reducing excitatory neurotransmission (Garcia et al., 2010, Nelson et al., 2002, Petrenko et al., 2014). Volatile anesthetics including isoflurane and sevoflurane are widely used general anesthetics and interact with multiple molecular targets (Franks, 2008, Hemmings et al., 2005). Isoflurane and sevoflurane can induce hyperactivity during anesthesia induction and recovery (Dahmani et al., 2010, Guo et al., 2017, Liang et al., 2017) and immobility at higher concentrations (Sonner et al., 2003). A variety of neuronal functions are bidirectionally modulated by volatile anesthetics (Chen et al., 2018, Yu et al., 2015, MacIver and Roth, 1987, MacIver and Roth, 1988). However, it is unclear how volatile anesthetics bidirectionally modulate neural excitability. Here, isoflurane at sub-anesthetic concentration induced hyperactivity *in vivo*. This is consistent with previous studies that sevoflurane can induce hyperactivity during induction and recovery (Costi et al., 2014, Guo et al., 2017, Liang et al., 2017). The isoflurane-induced hyperactivity was completely diminished in NALCN knockdown mice *in vivo*. Targeting NALCN channel in volatile anesthetic induction may prevent hyperactive behaviors, although other contribution of NALCN for respiratory modulation should be fully considered.

Isoflurane bidirectionally modulated neuronal excitability in acute brain slices, where isoflurane increased the spontaneous firing rate and depolarized RMP at sub-anesthetic concentrations but depressed the firing rate and hyperpolarized RMP at anesthetic concentrations. Because of the critical roles of NALCN and Na_v_ in modulating neuronal excitability and rising phase of APs, respectively (Bean, 2007, Hodgkin and Huxley, 1990), the differential effects of isoflurane on NALCN and Na_v_ may contribute to its bidirectional effects on neuronal excitability. NALCN currents and NALCN-mediated holding currents were activated by isoflurane starting from sub-anesthetic concentrations. NALCN contributes to the excitatory action of isoflurane because NALCN blocker Gd^3+^ diminished the increased firing rate. Inhibition of TTX-S Na_v_ can depress the firing rate even when NALCN is enhanced by SP. Isoflurane inhibited Na_v_ at anesthetic concentrations. Thus, isoflurane can depress firing rates and APs at anesthetic concentrations. The maintenance of RMP and the generation of APs also depend on multiple other ion channels including voltage-gated calcium channels and two-pore potassium channels (Patel et al., 1999, Suzuki et al., 2002, Westphalen et al., 2013). In this study, the observed effects of isoflurane on RMP can be a net interaction of isoflurane with multiple volatile anesthetic sensitive background channels (Storm, 1990). Notably, although only CA3 pyramidal neurons were recorded here, the bidirectional effect of isoflurane can be common because NALCN and TTX-S Na_v_ are widely expressed.

NALCN is highly expressed in neurons in the brain and spinal cord (Lu et al., 2007), and may be a molecular target for general anesthetics. NALCN can modulate the resting membrane potential of neurons and contributes to the rhythm of pacemakers (Cochet-Bissuel et al., 2014). Different volatile anesthetics may exert differential effects on NALCN (Cochet-Bissuel et al., 2014, Guan et al., 2000, Mir et al., 1997). Drosophila and nematode with UNC79 mutants are hypersensitive to the immobilizing effects of volatile anesthetics (Humphrey et al., 2007). Lwt/+ mice (mice with decreased level of UNC79, a key component of the NALCN protein complex) are resistant to anesthesia with isoflurane (Speca et al., 2010). Also, a case reported that a 3-year-old baby with a pathological mutation of NALCN showed hypersensitivity to volatile anesthetics (Lozic et al., 2016). All the above results indicate that NALCN complex is the underlying target of isoflurane to achieve the anesthesia state. In the present study, MAC of isoflurane for immobility was not influenced by NALCN knockdown in brain because spinal cord is the principal target of immobility induced by volatile anesthetics (Sonner et al., 2003). MAC of isoflurane for LORR was not affected by NALCN knockdown in brain neither. However, this result cannot exclude NALCN complex as the underlying target of isoflurane because the knockdown efficacy by NALCN-shRNA may not be high enough to exclude the effects of isoflurane on NALCN complex. Of note, the exact interaction between isoflurane and NALCN, UNC79, and UNC80 remains unclear. In the present study, isoflurane at ∼1.5 MAC did not enhance transfected rodent NALCN channel conductance *per se*, indicating isoflurane may enhance NALCN-mediated currents by UNC79 or UNC80 or other intracellular signaling.

Voltage-gated sodium channels (Na_v_) regulate neuronal excitability and provide an underlying presynaptic molecular target for volatile anesthetics (Hemmings, 2009, Herold and Hemmings, 2012, Ouyang et al., 2003). Here, isoflurane inhibited *I*_NaT_ and *I*_NaP_ at a physiological holding potential in hippocampal CA3 pyramidal neurons. *I*_NaT_ is fundamental for the initiation and propagation of APs (Hodgkin and Huxley, 1990), whereas *I*_NaP_ is able to increase excitabilities of neurons (Sittl et al., 2012). *I*_NaP_ has been shown to play important roles in the regulation of neuronal firing rates (Yue et al., 2005) and affect the behaviors of APs in the sub-threshold voltages especially (Lewis and Raman, 2014, Stafstrom, 2007). The effects of isoflurane on Na_v_ provide a neurophysiological mechanism consistent with previous reports that isoflurane reduces the excitability of CA3 pyramidal neurons by depressing APs (Westphalen and Hemmings, 2006, Wu et al., 2004). APs have been reported to play a pivotal role in synaptic transmission and plasticity of neurons (Deng et al., 2013). Isoflurane increases AP width and therefore reduces the frequency of presynaptic APs.

In conclusion, isoflurane activates NALCN conductance at sub-anesthetic concentrations and inhibits Na_v_ currents at anesthetic concentrations in hippocampal CA3 pyramidal neurons. These effects may contribute to the bidirectional modulation of neuronal excitability by isoflurane. The excitatory effects of isoflurane may promote behavioral hyperactivity during anesthesia induction. Targeting NALCN in volatile anesthetic induction may prevent hyperactive behaviors.

### Limitations of the Study

No selective blocker of NALCN is available, so we could not attempt to block isoflurane's effects by pharmacologically depressing these channels. We did not record the effects of isoflurane on neuronal firing rate in the NALCN knockdown mice after behavioral tests. In wild-type mice, ∼20%–30% hippocampal neurons produced spontaneous discharge and sub-anesthetic isoflurane increased the discharge frequency. After knockdown of NALCN, no hippocampal neuron produced spontaneous discharge because of decreased neuronal excitability. Meanwhile, the effects of volatile anesthetics on ion channels can differ between agents. We did not know whether other commonly used volatile anesthetics such as sevoflurane would produce similar effects.

## Methods

All methods can be found in the accompanying Transparent Methods supplemental file.

## Supplemental Information

Document S1. Transparent Methods and Figure S1
