# Supplementary material for: The General Anesthetic Isoflurane Bilaterally Modulates Neuronal Excitability
Source: iScience. 2019 Dec 10;23(1):100760. doi: 10.1016/j.isci.2019.100760 (PMC6956953; doi:10.1016/j.isci.2019.100760)
Supplement: Document S1. Transparent Methods and Figure S1 [file mmc1.pdf]

ISCI, Volume *23*

Supplemental Information

The General Anesthetic Isoflurane Bilaterally Modulates Neuronal Excitability

Mengchan Ou, Wenling Zhao, Jin Liu, Peng Liang, Han Huang, Hai Yu, Tao Zhu, and Cheng Zhou

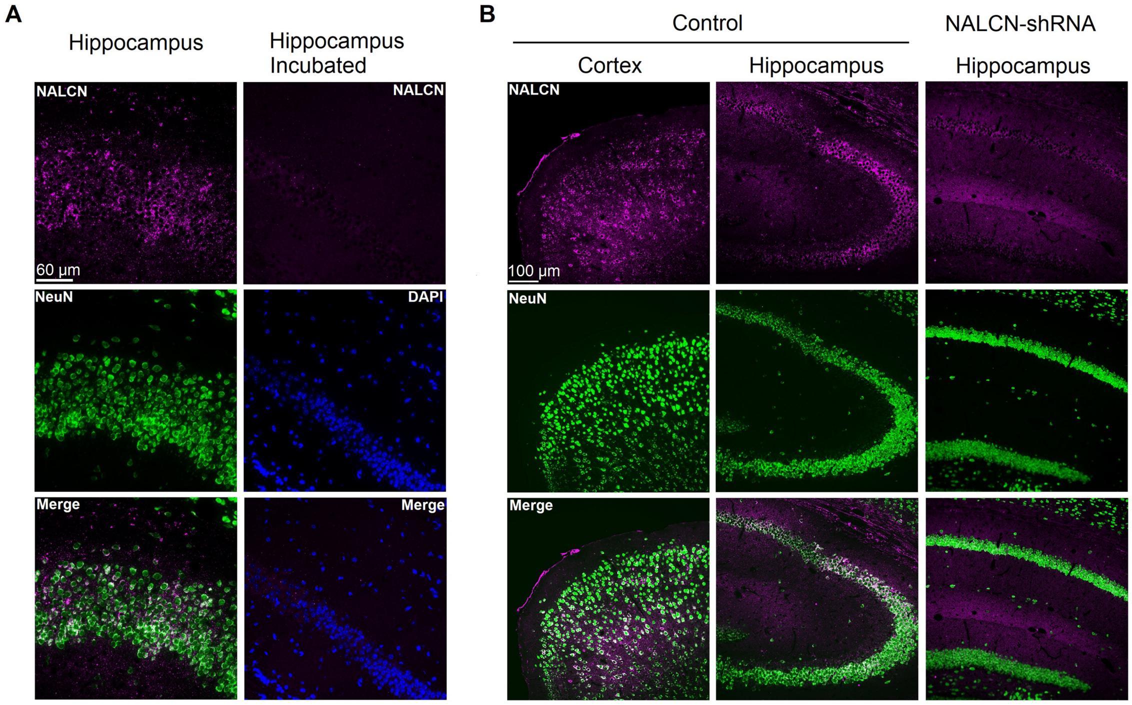

Supplementary Figure S1. Specificity of NALCN antibody was validated in cortex and hippocampus of mice, Related to Figure 1.

A: NALCN was stained in mice hippocampus and well merged with NeuN (left). NALCN positive fluorescence was almost diminished in mice hippocampus when the antibody pre-incubated with NALCN antigen (right). **B:** The expression profiles of NALCN channel in cortex and hippocampus from control mice (left) and NALCN knockdown mice (right) were detected by immunofluorescence staining.

Transparent Methods

Animals

All experiments were conducted in compliance with the Medical Laboratory Animal Management Rules (Chinese Health and Family Planning Commission) and the Animal Ethics Committee of Sichuan University (Chengdu, China). Neonatal (7-12 days, 37 males and 42 females, 79 in total) and adult (~8 weeks, 8 males and 8 females) C57BL/6 mice were used. All the mice were kept under standard experimental conditions (temperature of 22 ± 2°C, humidity of 40%-70% and a 12/12h light/dark cycle) with free access to food and water.

Immunofluorescence staining

Animals were anesthetized with ketamine/xylazine (60/10 mg/kg), and then transcardially perfused with 4% paraformaldehyde. Brains were removed and put into 4% paraformaldehyde solution overnight, followed by 30% sucrose for one day. Transverse sections of brains (12 μm) were cut using a freezing microtome (CM1850; Leica, Buffalo Grove, IL, USA). Sections were incubated at 4°C overnight with primary antibodies, namely: NALCN (1:400, rabbit, ASC-022, Alomone Labs, Israel), NeuN (1:400, mouse, MAB377, Millipore, Temecula, CA, USA). Then they were incubated with secondary antibodies for 2 hours: Alexa Fluor 647 goat anti-mouse (115-605-003), Alexa Fluor 488 goat anti-rabbit (111-545-003) (Jackson ImmunoResearch, West Grove, PA, USA 19390). Fluorescent image acquisition was performed using Zeiss AxioImager Z.2. Specificity of the NALCN primary antibody was validated as presented in supplementary **Figure S1**. Briefly, NALCN primary antibody was pre-incubated with the antigen (available from the manufacturer) and also compared in NALCN knockdown mice.

Preparation of mouse hippocampal slices

C57BL/6 mice at 7-10 postnatal days were anesthetized with ketamine/xylazine (60/10 mg/kg) and decapitated. The brain was rapidly removed and put into ice-cold oxygenated (95%O_2/5%CO_2) sucrose-substituted dissecting solution

containing (in mM): 260 sucrose, 3 KCl, 5 $MgCl_2$, 1 $CaCl_2$, 1.25 NaH_2PO_4, 26 $NaHCO_3$, 10 glucose, and 1 kynurenic acid. Transverse hippocampal slices (270 μm) were cut using a vibratome (VT1000 A; Leica), incubated for 30 min at 37°C and then at room temperature (24-26°C) in incubation solution containing (in mM): 130 NaCl, 3 KCl, 2 $MgCl_2$, 2 $CaCl_2$, 1.25 NaH_2PO_4, 26 $NaHCO_3$, and 10 glucose. The incubation solutions were aerated with 95%O_2/5%CO_2. After incubation, hippocampal slices were mounted in the recording chamber for electrophysiological recordings at room temperature (Yamada-Hanff and Bean, 2015).

NALCN cDNA construct and transfection

Human embryonic kidney (HEK293T) cells were used. Rat NALCN (NALCN; GenBank accession no. NM_153630.1) (Lu et al., 2007 and 2010) cDNA was constructed into pcDNA 3.0 plasmid and tagged with eGFP (Tsingke Biological Technology, Beijing, China). HEK293T cells were maintained in Dulbecco's Modified Eagle's Medium (DMEM) containing 10% fetal bovine serum (FBS), 1% antibiotic-antimycotic and supplemented with 1 mM sodium pyruvate. All constructs were transfected using Lipofectamine 2000 (Invitrogen, US) according to the manufacturer's instructions. After transfection, the cells were cultured for another 24 h before recording.

Patch-clamp recording

Hippocampal slices were placed in a recording chamber submerged in a continuously perfused external solution (2 ml/min), and bubbled with 95%O_2/5%CO_2. Pyramidal neurons in the hippocampal CA3 region were directly visualized and identified by their shape and size. Electrophysiological recordings were conducted using an Axopatch 200B amplifier and Digidata1440 digitizer linked to a computer running pClamp 10.2 software (Molecular Devices, Sunnyvale, CA, USA). Currents were sampled at 20 kHz and filtered at 5 kHz. Recordings were performed in either cell-attached or whole-cell configurations. Voltage-clamp

recordings were established to record spontaneous firing rate and holding currents. Holding currents and conductance were monitored over time by delivering –60 mV voltage steps every 15 s. The current-voltage (I-V) relationship of the isoflurane-sensitive current, determined by subtracting current responses to positive and negative voltage steps (–60 to +30 mV), was obtained in the presence of isoflurane. Pipette internal solution was used for recordings of spontaneous firing rate (cell-attached) and APs (whole-cell) contained the following (in mM): 120 KCH_3SO_3, 4 NaCl, 1 $MgCl_2$, 0.5 $CaCl_2$, 10 HEPES, 10 EGTA, 3 Mg-ATP, and 0.3 GTP-Tris, pH 7.3. Instead, a Cs-based internal solution was used to record the Na_v currents, which contained the following (in mM): 110 CsF, 9 NaCl, 1.8 $MgCl_2$, 4 Mg-ATP, 0.3 Na-GTP, 0.09 EGTA, 0.018 $CaCl_2$, 9 HEPES, and 10 TEA-Cl, pH 7.38. The external solution was the same as the incubation solution, but added 25 mM TEA-Cl. Tetrodotoxin-sensitive (TTX-S) Na_v currents were confirmed by subtraction after application of 500 nM TTX. Series resistance was compensated by ~70%-75%, and data were rejected when series resistance exceeded 15 MΩ. APs were recorded under current-clamp mode. Bicuculline (10 μM) and picrotoxin (100 μM) were added to block possible synaptic and/or extra synaptic GABAergic inputs.

Virus injection

Juvenile C57BL/6 mice (P21) were anesthetized with ketamine/xylazine (60/10 mg/kg) and fixed in a stereotaxic frame. The skull of mice was drilled and a pipette filled with pAAV2-H1-shRNA-(NALCN)-CAG-eGFP or pAAV2-scrambled-CAG-eGFP virus (2 x 10^{13} TU/ml) was injected bilaterally into the lateral ventricles (0.3 mm caudal to Bregma; ± 1 mm lateral to the midline; and 2.0 mm ventral to the surface). The speed of injection was 0.5 μL/min with total volume of 1 μL for each side. The NALCN and scrambled shRNA were selected as previously described (Shi et al., 2016): AAGATCGCACAGCCTCTTCAT; GCTCAGTACGATCATACTCAC (scrambled).

Behavioral tests

Activity of mice during anesthesia induction was recorded as previously described (Liang et al., 2017). Briefly, four weeks after injection of virus, the mice were placed in a transparent plastic cylinder (30 x 10 cm) with airflow of 3 L/min. A white heating pad was put under the cylinder and kept at 37°C. The first 3 min in air was recorded as baseline, then isoflurane at 0.2%, 0.5% and 1.3% was introduced and each concentration was maintained for 2 min. The concentration of isoflurane was monitored by a gas detector (Datex-Ohmeda, Louisville, CO, USA). The movement tracks of mice were recorded by a camera (Canon, Legria, Tokyo, Japan, HF R706) and analyzed by behavioral tracking software (Smart 2.5, Panlab, DL Naturgene Life Science, Inc., Beijing, China). After a 5-day washout, minimum alveolar concentration (MAC) of isoflurane for loss of righting reflex (LORR) and immobility (Tail-clamping) were determined with the same system. MAC for LORR was the mean concentration of isoflurane that induce loss of righting reflex and MAC for immobility was the mean concentration of isoflurane that induce unresponsive state to tail-clamping for 60 s.

Quantitative real-time polymerase chain reaction (qRT-RCP)

NALCN mRNA was measured by qRT-PCR. The cortex and hippocampus of mice were dissected. Total RNAs was extracted using the Eastep® Super RNA extraction kit (Promega, Shanghai, China). The synthesis of cDNA was executed with a GoScript™ Reverse Transcription Kit (Promega, Shanghai, China). Then the cDNA was used as templates and assayed to quantify the NALCN expression level using GoTaq® qPCR Master Mix (Promega, Shanghai, China) and specific primers (Sangon Biotech, Shanghai, China) according to the manufacturer's protocol. The forward and reverse primers of NALCN are (5'-3') GTCCTGACGAATCTCTGTCAGA and CTGAGATGACGCTGATGATGG, respectively. The GAPDH gene was used as an internal control. The PCR conditions were as following: 3 min at 95°C; 40 cycles: 15 s, 95°C; 30 s, 55°C; 30 s, 72°C.

Statistical analysis

Electrophysiological data were analyzed using Clampfit 10.0 software (Molecular Devices), Graph-Pad Prism 7 (Graph-Pad Software, San Diego, CA, USA). All data are presented as mean ± SEM. Statistical analysis was performed using SPSS version 22.0 (SPSS Inc., Chicago, Illinois, USA) and GraphPad Prism 7. A paired or unpaired t-test, or a one-way repeated-measures ANOVA followed by the Newman-Keuls multiple-comparisons test were used as appropriate. Statistical significance was set at $P < 0.05$.
